# The pattern of color change in small mammal museum specimens: is it independent of storage histories given museum-specific conditions?

**DOI:** 10.1186/s13104-018-3544-x

**Published:** 2018-07-03

**Authors:** María Leonor Sandoval Salinas, José D. Sandoval, Elisa M. Colombo, Rubén M. Barquez

**Affiliations:** 1 Instituto de Investigación en Luz, Ambiente y Visión-ILAV, Universidad Nacional de Tucumán-UNT, Consejo Nacional de Investigaciones Científicas y Técnicas-CONICET, Av. Independencia 1800, 4000 San Miguel de Tucumán, Tucumán Argentina; 2 Programa de Investigaciones de Biodiversidad Argentina-PIDBA, Facultad de Ciencias Naturales e Instituto Miguel Lillo-FCNeIML, Universidad Nacional de Tucumán-UNT, Miguel Lillo 205, 4000 San Miguel de Tucumán, Tucumán Argentina; 3 Departamento de Luminotecnia, Luz y Visión-DLLyV, Facultad de Ciencias Exactas y Tecnología-FaCET, Universidad Nacional de Tucumán-UNT, Av. Independencia 1800, 4000 San Miguel de Tucumán, Tucumán Argentina

**Keywords:** *Akodon budini*, Objective color measurement, Rodentia, Sample age, Spectroradiometry

## Abstract

**Objective:**

Determination of color and evaluating its variation form the basis for a broad range of research questions. For studies on taxonomy, systematics, etc., resorting to mammal specimens in museum collections has a number of advantages over using field specimens. However, if museum specimens are to be for studying color, they should accurately represent the color of live animals, or we should understand how they differ. Basically, this study addresses this question: How does coat color vary when dealing with specimens of *Akodon budini* (Budin’s grass mouse, Thomas 1918), stored in one museum collection for different periods of time?

**Results:**

We measured color values through a spectroradiometer and a diffuse illumination cabin and used the reflectance values in the form of CIELab tri-stimulus values, considering CIE standard illuminant A. We observed that there is a relationship between specimen storage antiquity and pelage color and it seems that it is general for at least a number of small mammals and this could indicate a universal phenomenon across several mammal species and across several storage conditions. Our results, as others, emphasize the importance of considering storage time, among other circumstances, in research studies using mammal skins and where color is of importance.

**Electronic supplementary material:**

The online version of this article (10.1186/s13104-018-3544-x) contains supplementary material, which is available to authorized users.

## Introduction

Since the stored specimens in the biological and paleobiological collections have associated core data that are recognized as fundamental for discipline-specific research [1, 2], as well as for broader global issues and public initiatives [3], existing collections in Natural History museums are often the basis of numerous studies in taxonomy, systematics, ecology, biogeography, management, conservation, etc. [1, 4–12], and studies based on biological museum specimens are to a large degree the basis of our understanding of the diversity of life on Earth [6, 13]. In this framework, determination of color and evaluating its variation form the basis for a broad range of research questions. Accordingly, color itself is often the primary target of museum-based studies.

Resorting to mammal specimens in museum collections has a number of advantages over using field specimens, given the taxonomic, geographic, and temporal range of samples, which is near impossible to achieve by sampling in the wild [14]. Particularly for terrestrial vertebrates, historic specimens stored in collections have been productively used in quantitative and comparative studies [15]. Because research involving the study of coat color in mammals is better if it includes a wide-range sample, those studies often rely on specimens stored in museums.

If museum specimens are to be for studying color, they should accurately represent the color of live animals, or we should understand how they differ [16]. There have been numerous investigations related to temporal changes in coloration of bird specimens in museum collections [e.g., 14, 16–22]. In all cases, the results showed substantial differences in the measured reflectance spectra of newer and older specimens. Meanwhile, variations of the coat color of mammal specimens in relation to storage time have not been studied, with few exceptions [e.g., 23, 24]. As other colors of the animals, coat color is susceptible to deterioration with time due to chemical or structural degradation of pigments that may result in the alteration of their optical properties [15]. Besides, it has been found that color of museum specimens other than mammals is influenced by specimen preparation, preservation process, and storage histories [14, 22]. This effect has been acknowledged explicitly as a possible limiting factor in research on collection material [22–24]. Therefore, it seems reasonable to ask to what extent the color of museum mammal specimens may be considered an acceptable representation of the color of live animals [20, 22].

Our main goal was to apply an objective color measurement methodology to the detection, if any, of patterns of changes in the fur color of specimens of a small mammal species, *Akodon budini* (Budin’s grass mouse, Thomas 1918), in relation to the storage time (given the museum-specific preparation, preservation and storage conditions). By doing this, we aim to assess the use of museum collections in research on mammal color.

## Main text

### Materials and methods

We studied the same *Akodon budini* series previously studied for other purposes [25, 26]. For analyses we used skins from 54 adult specimens (Additional file 1). All specimens have been skinned and stored with the same methods [27].

We matched sex, collection dates (month and year) and occurrence localities (recorded from the information on specimen tags) to each specimen color data. Collection year ranged from 1970 to 2008.

As we did in our previous studies [25, 26] we measured color data (Additional file 2) through a Photo Research PR715 spectroradiometer and the SpectraWin 2 software, and inside a diffuse illumination cabin. This instrument measures the sample spectral reflectance. From the reflectance values, tri-stimulus values, which may be defined according to different systems [28], can be derived [29]. We worked with the CIE (Commission Internationale de L’Eclairages, or International Commission on Illumination) Lab color system (Additional file 2). We selected the CIE Lab color space [29] because it is based on the human visual model, and therefore includes all of the colors perceived by humans [30], and it is more appropriate for the biological aims of this study [31]. For calculation of these tri-stimulus values, it is imperative the use of standard conditions for observation, which includes the determination of one of the several types of standard illuminants defined by the CIE. We worked with the CIE standard illuminant A, which represents typical, domestic, tungsten-filament lighting.

We recorded the chromatic coordinates of five points (neck, upper back, middle back, lower back, and rump) over the dorsal middle line of each specimen (Additional file 3).

We used the computer statistical package INFOSTAT (2013 version) to conduct all statistical analyses over color data. We used Principal Component Analysis (PCA) to describe the association between the color variables, the independent variables, and each of the observations [32–36]. We then used general linear models of analysis of variance (ANOVAs) to examine relationships between color data and sample antiquity, and to explore the relationships between color data and sex, collection season, and sampling location. We included specimen identity as a random effect. We included in the analysis all interaction terms. We then proceed to model simplification and removed each non-significant (p > 0.05) interaction term. We used as post hoc test the Fisher’ LSD statistic with Bonferroni correction. Significance was accepted when p < 0.05. Finally, we report results related to those parameters that explain substantial variation in each dependent variable as determined by model fitting.

### Results

In Additional file 4, we present plots of the evolution of the color variables against their antiquity.

#### PCA (Table 1, Fig. 1)

**Fig. 1:**
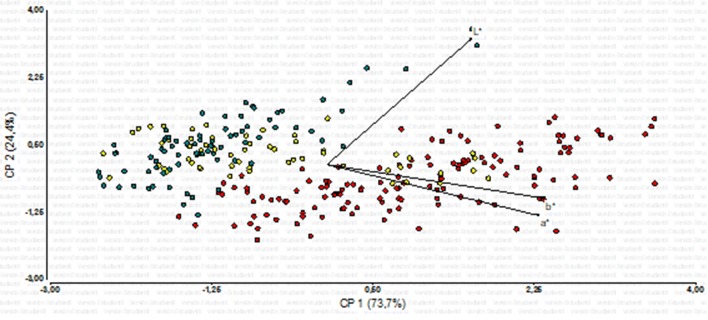
Principal Component Analysis: L*, a*, and b* values obtained for the five dorsal measuring points from 54 *Akodon budini* specimens. Dots were painted according to sample age. Red: specimens collected in the 1970s; yellow: specimens collected in the 1990s; blue: specimens collected in the 2000s

In Fig. 1 we painted dots according to their sample age. With respect to PC1, overlapping of the three dot clouds is extensive; however, most dots corresponding to specimens collected in the 1970s are located toward the right, in the direction of increasing values of a* and b*, and to a lesser extent of L*. The opposite applies to dots corresponding to specimens collected in the 2000s: they are located to the minimum values of a* and b* (and to a lesser extent of L*). Moreover, 1970s’ dots are highly displaced relative to the two other dot clouds: no 1990s’ or 2000s’ dots reaches the values of the 1970s’ dots located to the right. With respect to PC 2, while overlapping of the three dot clouds is again extensive, most 1970 dots are located down, in the direction of (moderate) increasing values of a* and b*, and decreasing values of L*. The opposite applies to the 2000 dots, which are located upwards, in the direction of (moderate) decreasing values of a* and b*, and increasing values of L*. In any case, dots corresponding to specimens collected in the 1990s have an intermediate position.

**Table 1:** Principal Component Analysis (PCA) of color variables

A.		
Variables	e1	e2
L*	0.43	0.90
a*	0.63	− 0.36
b*	0.65	− 0.24

B.			
Lambda	Value	Proportion	Cum prop
1	2.21	0.74	0.74
2	0.73	0.24	0.98
3	0.06	0.02	1.00

Results from the PCA of color variables L* (level of lightness), a* and b* (chromaticity) are shown. A. Eigenvectors1, 2, and 3: e1 and e2, respectively. B. Eigenvalues

#### Anova (Table 2)

Color data measured in specimens stored for different periods of time are significantly different. We also found significant interactions between sample antiquity and measuring point [26] (Table 2). These results was found for two of the color variables, those that account for chromaticity (a* and b*; Table 2). This indicates that chromaticity, but not lightness (L*), varies in relation to measuring points and that this variation occurs at significantly different rates in different antiquity samples.

**Table 2:** Analysis of variance (ANOVA) of color variables

	L*		a*		b*	
	F value	p value	F value	p value	F value	p value
Sample antiquity	2.87	0.0672	38.72	*< 0.0001*	20.46	*< 0.0001*
Sex	0.06	0.8130	0.07	0.7855	0.17	0.6852
Collection season	13.39	< 0.0001	11.07	0.0001	5.01	0.0109
Sampling location	3.34	0.0745	5.39	0.0249	5.54	0.0230
Measurement point	5.68	0.0011	0.72	0.5409	13.08	< 0.0001
Antiquity × point	1.07	0.3814	6.47	*< 0.0001*	9.49	*< 0.0001*
Sex × point	0.57	0.6352	5.02	0.0025	3.23	0.0246
Season × point	2.47	0.0267	8.72	< 0.0001	2.98	0.0091
Sampling location × point	2.51	0.0617	6.29	0.0005	10.73	< 0.0001

Statistically significant p values, in relation to the sample antiquity (and its interaction with measurement point), are indicated in italics

Results from the ANOVA in which the variation in L*, a*, and b* of the dorsal fur color of *Akodon budini* specimens in relation to sample antiquity (and other variables presented and discussed elsewhere [29, 30]) are shown. Separate analyses were performed with L*, a*, or b* as dependent variable

### Discussion

Similar to what happens with other biological pigments, the melanin-based colors may change over time due to chemical or structural degradation, from the time the mammal specimens are collected in the field, and during their storage in museum collections. Since museum collections of mammal specimens provide an important resource for evolutionary investigations of mammal phenotypic diversity, we examined this essential source of variation in physical measurements of a given small mammal species coloration and addressed the impact of time since collection on pelage color. We found that there is a relationship between specimen storage antiquity and pelage color in the *Akodon budini* studied sample: specimens get yellower and redder with storage antiquity. It could be said that the observed color change corresponds to the natural aging of the coat and its pigments, which is determined by the prevailing storage conditions.

Studies of changes in plumage coloration across storage have sometimes deal with melanin-based colors. Particularly, [22] examined not only color variations in carotenoid-based pigments of the feathers (as many studies dealing with bird coloration) but also the black melanin-based color. These results are relevant since they can be compared to the melanin-based color of mammals [24]. According to [22], feathers with melanin-based colors tend to have higher red chroma in older specimens. They speculated that eumelanin (the one of the two melanin pigments that is responsible for dark colors) may be more susceptible to degradation than pheomelanin (the other melanin pigment, responsible for yellow and red colors). If this is the case, eumelanin degradation over time led to reduced proportions of this pigment in the feathers, resulting in feathers slightly reddish [22]. Similarly, [23] found that the coat color of a red bat species (*Lasiurus borealis*) vary significantly with the antiquity of the specimens, with older specimens having redder shades of pelage than newer ones. Considering those results, it seems likely that the pelage of *Akodon budini* may have suffered breakdown of eumelanin pigment in the hair fibers, as suggested by [22], which would result in greater proportions of the redder pheomelanin pigment and a redder overall coat color in older specimens [24].

This eumelanin–pheomelanin relative proportions hypothesis may also be the explanation of why the interaction of Antiquity × Point is highly significant for a* and b* color variables (Table 2). As we established in our previous work [26], color determinations strongly depend on the measuring point, even on the same body area. In [26], for most studied specimens, the values of the variables a* and b* decreased in a neck-rump direction in specimens of the same age: those specimens are naturally redder and yellower to their necks, which may indicate that near their heads, hairs have relatively more pheomelanin. If this is the case, eumelanin degradation will be more evident near their tails.

Assessing the limitations of using museum specimens for characterizing the pelage color of small mammals implies to reflect on the usefulness of the study of museum specimens for characterization, identification, and discrimination of different taxonomic units based on color. Knowledge and understanding of color variation with specimens storage time in a given museum collection could allow researchers to correctly interpret the pelage color of specimens with different antiquities and to evaluate the differences in a framework of adequate interpretation, in order to precisely determine the limits of the color variation ranges of a particular taxonomic entity and to establish the usefulness of such ranges in identifying different taxa.

Since our and other studied mammal specimens are housed in Mammal Collections with very different storage conditions, it seems that there is a relationship between specimen storage antiquity and pelage color that is general for at least a number of small mammals and this could indicate a universal phenomenon across several mammal species and across several storage conditions. Our study does not intend to take away support from the use of museum specimens to examine intra or inter-specific pelage coloration in mammals. Our results, as those of [23] and [24], emphasize the importance of considering storage time, among other already mentioned circumstances, in research studies using mammal skins and where color is of importance.

## Limitations

Limitations include having worked with a sample of a single small mammal species from a single museum. However, our results aim to complement those previously obtained for other small mammal species from other museums. Even more, a more inclusive study is in process, in which numerous species coming from several museums (one of Argentina and 3 of the USA) are included.

Also we will use this study as an initial point of a long-term study.

## Additional files

**Additional file 1.** Examined specimens of the species included in this study. Specimens are individualized by the acronym of the collection or collector and the corresponding number in the collection or collector catalog. CML is the Spanish acronym of the Lillo Mammal Collection -CML- (Natural Sciences Faculty and Miguel Lillo Institute, National University of Tucuman, Argentina), and LIF and LT-RMB are collector acronyms. Specimens are arranged according to their sample antiquity, geographical origin (political province), collection season, and sex.

**Additional file 2.** Color basics: A summary of the general nature of color.

**Additional file 3.** Photograph of a specimen of *Akodon budini* placed within the diffuse illumination cabin showing the five points over the dorsal body region, namely the neck, upper back, middle back, lower back, and rump, where we measured the pelage color (see “Materials and Methods”). Figure from Anais da Academia Brasileira de Ciências (2016) (Annals of the Brazilian Academy of Sciences) Printed version ISSN 0001-3765 / Online version ISSN 1678-2690 http://dx.doi.org/10.1590/0001-3765201620150004 www.scielo.br/aabc.

**Additional file 4.** Plots of the evolution of the color variables, L*, a*, and b*, against the year of sampling.

## Abbreviation

CIE: Commission Internationale de L’Eclairages, or International Commission on Illumination
