# Supplementary material for: The pattern of color change in small mammal museum specimens: is it independent of storage histories given museum-specific conditions?
Source: BMC Res Notes. 2018 Jul 3;11:424. doi: 10.1186/s13104-018-3544-x (PMC6029030; doi:10.1186/s13104-018-3544-x)
Supplement: Supplementary file 1 — Additional file 1. Examined specimens of the species included in this study. Specimens are individualized by the acronym of the collection or collector and the corresponding number in the collection or collector catalog. CML is the Spanish acronym of the Lillo Mammal Collection -CML- (Natural Sciences Faculty and Miguel Lillo Institute, National University of Tucuman, Argentina), and LIF and LT-RMB are collector acronyms. Specimens are arranged according to their sample antiquity, geographical origin (political province), collection season, and sex. [file 13104_2018_3544_MOESM1_ESM.docx]

Additional file 1

Examined specimens of the species included in this study. Specimens are individualized by the acronym of the collection or collector and the corresponding number in the collection or collector catalog. CML is the Spanish acronym of the Lillo Mammal Colection -CML- (Natural Sciences Faculty and Miguel Lillo Institute, National University of Tucuman, Argentina), and LIF and LT-RMB are collector acronyms. Specimens are arranged according to their sample antiquity, geographical origin (political province), collection season, and sex.

*Akodon budini*

1970

JUJUY:

Winter

Males (n=10)

CML 1776, CML 1789, CML 1790, CML 1791, CML 1792, CML 1793, CML 1794, CML 1797, CML 1798, CML 1807

Females (n=4)

CML 1799, CML 1800, CML 1801, CML 1806

Summer

Males (n=10)

CML 1738, CML 1739, CML 1740, CML 1741, CML 1743, CML 1747, CML 1748, CML 1749, CML 1750, CML 1751

Females (n=3)

CML 1744, CML 1745, CML 1746

1990

JUJUY:

Winter

Males (n=5)

CML 4603, CML 4604, CML 4608, CML 4611, CML 4612

Females (n=3)

CML 4605, CML 4606, CML 4607

Midseason

Males (n=1)

CML 4613

SALTA:

Midseason

Males (n=2)

CML 5524, CML 5526

Indeterminate sex (n=1)

CML 5529

2000

JUJUY:

Midseason

Males (n=5)

LIF 743, LIF 749, LIF 750, LT-RMB 114, LT-RMB 120

Females (n=4)

LIF 744, LT-RMB 112, LT-RMB 113, LT-RMB 119

Winter:

Females (n=2)

CML 8978, CML 8980

SALTA

Winter

Males (n=4)

LIF 810; LIF 818; LIF 824; LIF 827
