# Supplementary material for: The pattern of color change in small mammal museum specimens: is it independent of storage histories given museum-specific conditions?
Source: BMC Res Notes. 2018 Jul 3;11:424. doi: 10.1186/s13104-018-3544-x (PMC6029030; doi:10.1186/s13104-018-3544-x)
Supplement: Supplementary file 2 — Additional file 2. Color basics: A summary of the general nature of color. [file 13104_2018_3544_MOESM2_ESM.docx]

Additional file 3

*Color basics:*

A summary of the general nature of color.

Color is one of the fundamental characteristics defining our conception of the world around us, our appreciation of it, our positioning in it. The assessment of this sensorial characteristic is a crucial task that allows us to make decisions and take actions.

Color is basically specified by the geometry and spectral distributions of three elements: light source, reflection (or transmission) characteristics of the object, and visual sensitivity of the observer. In 1931, the CIE (Commission Internationale de l’Eclairage) stated a definition for each of these. These definitions were an attempt to simulate the human color perception, based on a 2° field of view, a set of primaries (red, green, blue; named RGB) and a standard observer, stating color-matching functions (CMFs [1]). Then, these RGB CMFs were transformed mathematically into xyz CMFs, yielding a new set of ‘artificial’ primaries X, Y, Z that can be used to compute the luminance of a color under analysis, according to Grassman’s additive law.

From here, any color can be described by a combination of these primaries.

In this context, the sensory properties of the objects around us (as the appearance and color of their surfaces, which are among the first visually evaluated parameters and consequently determine the observer relationship between objects and actors in the scene being observed) could be estimated [2].

A description of an object color in terms of RGB refers to the proportion of the intensity of three well defined primary lights (red, green, blue) in the composition of that color, based on the additive synthesis which is in the very beginning of human perception.

The CIE system of colorimetry (XYZ, derived from RGB) is a system of colour specification. It allows us to predict matching conditions even with spectrally dissimilar stimuli, but not to predict color appearance. It has two main limitations: the chromaticity of a perfect diffuser changes when the color of the illumination changes; and it is markedly non-uniform: the Euclidean distance between two points in XYZ space does not represent the same perceptual change everywhere in that space [3].

A major advance was made in 1976 with the introduction of the CIE L* a* b* (or CIELAB) color specification system. This is a non linear transformation of XYZ values that partially solved the problems of color appearance and color differences provoked by the lack of perceptual uniformity of the former system.

Both systems share the same fundamental principles, i.e.:

- Color is a sensation that results from the interaction of a light source, an object, and a observer (a light source illuminates an object; an object modifies light, and reflects –or transmits- it to an observer; an observer senses the reflected light);

- Tristimulus values are coordinates of color sensation, computed from the CIE data.

But, the 1976 CIE L* a* b* system offers important advantages:

- It is more perceptually uniform;

- It is based on the accepted and useful theory of opponent colors.

The CIE L* a* b* color space is the most widely used method for measuring and ordering object color. It is employed throughout the world by those who have something to do with color of textiles, inks, paints, plastics, papers, printed materials and other objects (in our case, the color of the fur of mammals).

1. Mortimer RJ, Varley TS. Quantification of colour stimuli through the calculation of CIE chromaticity coordinates and luminance data for application to in situ colorimetry studies of electrochromic materials. Displays. 2011;32:35-44.

2. Saldaña E, Siche R, Huamán R, Luján M, Castro W, Quevedo R. Computer vision system in real-time for color determination on flat surface food. Scientia Agropecuaria. 2013;4:55-63.

3. Westland S. Review of the CIE system of colorimetry and its use in dentistry. J Esthet Restor Dent. 2003;15:S5–S12.
