# Supplementary figures and images for: The pattern of color change in small mammal museum specimens: is it independent of storage histories given museum-specific conditions?
Source: BMC Res Notes. 2018 Jul 3;11:424. doi: 10.1186/s13104-018-3544-x (PMC6029030; doi:10.1186/s13104-018-3544-x)

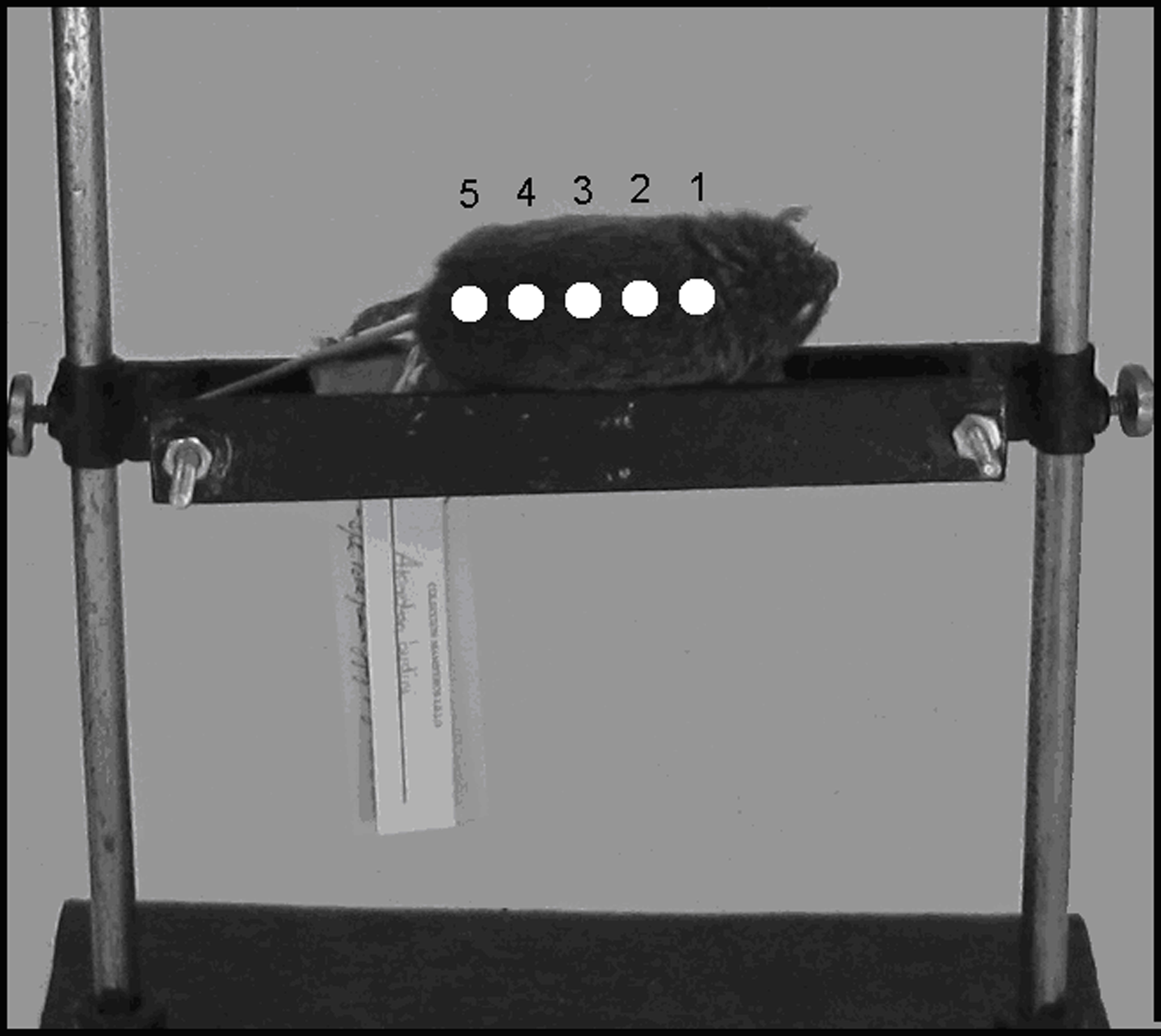

Supplement: Supplementary file 3 — Additional file 3. Photograph of a specimen of Akodon budini placed within the diffuse illumination cabin showing the five points over the dorsal body region, namely the neck, upper back, middle back, lower back, and rump, where we measured the pelage color (see “Materials and Methods”). Figure from Anais da Academia Brasileira de Ciências (2016) (Annals of the Brazilian Academy of Sciences) Printed version ISSN 0001-3765 / Online version ISSN 1678-2690 http://dx.doi.org/10.1590/0001-3765201620150004 www.scielo.br/aabc. [file 13104_2018_3544_MOESM3_ESM.tif]
