# Supplementary material for: The pattern of color change in small mammal museum specimens: is it independent of storage histories given museum-specific conditions?
Source: BMC Res Notes. 2018 Jul 3;11:424. doi: 10.1186/s13104-018-3544-x (PMC6029030; doi:10.1186/s13104-018-3544-x)
Supplement: Supplementary file 4 [file 13104_2018_3544_MOESM4_ESM.docx]

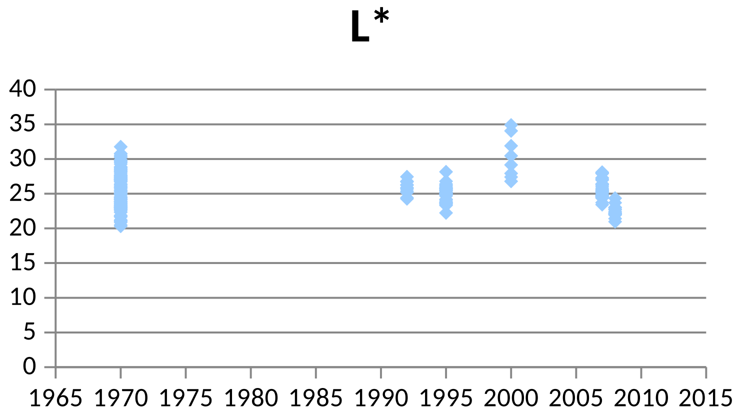
L*
40
35
30
25
20
15
10
5
0
1965 1970 1975 1980 1985 1990 1995 2000 2005 2010 2015

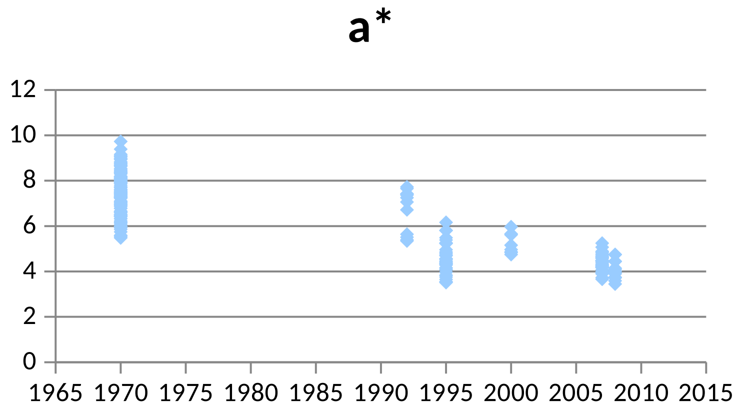
a*
12
10
8
6
4
2
0
1965 1970 1975 1980 1985 1990 1995 2000 2005 2010 2015

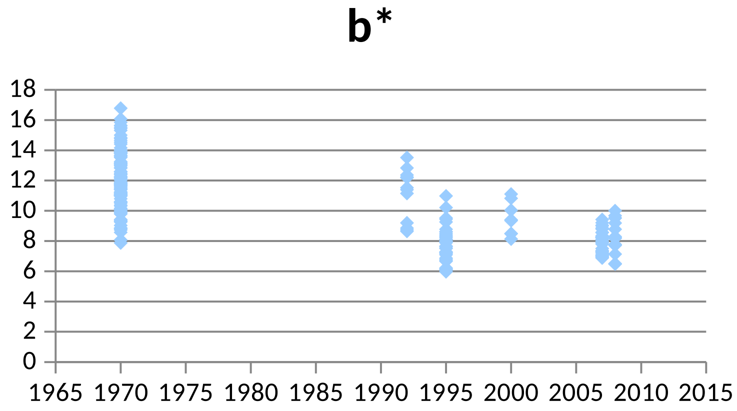
b*
18
16
14
12
10
8
6
4
2
0
1965 1970 1975 1980 1985 1990 1995 2000 2005 2010 2015
